# Supplementary material for: Splice-Junction-Based Mapping of Alternative Isoforms in the Human Proteome
Source: Cell Rep. Author manuscript; Available in PMC 2020 Jan 15. (PMC6961840; doi:10.1016/j.celrep.2019.11.026)
Supplement: 2 [file NIHMS1546469-supplement-2.zip › DF1/PXD010154/Ovary/Ovary_22_NSUN2_YRCPPPCPEGFVIANDVDNKR.pdf]

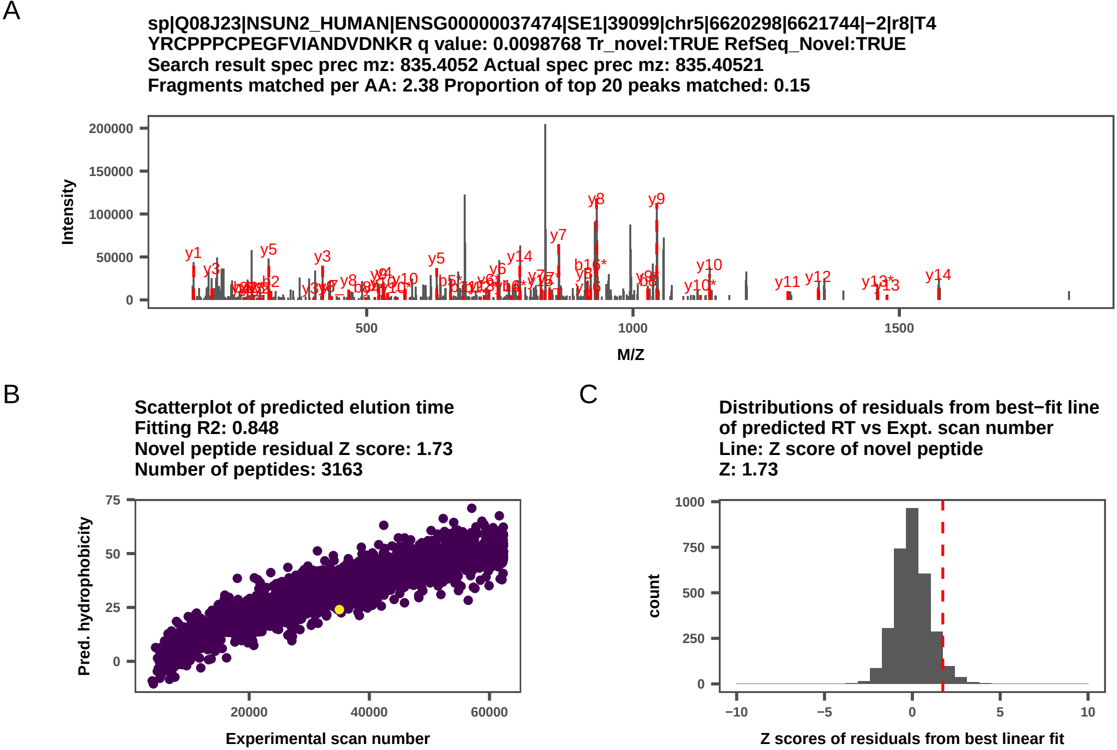
A
sp|Q08J23|NSUN2_HUMAN|ENSG00000037474|SE1|39099|chr5|6620298|6621744|−2|r8|T4
YRCPPPCPEGFVIANDVDNKR q value: 0.0098768 Tr_novel:TRUE RefSeq_Novel:TRUE
Search result spec prec mz: 835.4052 Actual spec prec mz: 835.40521
Fragments matched per AA: 2.38 Proportion of top 20 peaks matched: 0.15
Intensity
200000
150000
100000
50000
0
500
1000
1500
M/Z
y1
y3
y5
y3
y5
y14
y7
y8
y9
b16*
y10
y10*
y11
y12
y13
y14
B
Scatterplot of predicted elution time
Fitting R2: 0.848
Novel peptide residual Z score: 1.73
Number of peptides: 3163
Pred. hydrophobicity
75
50
25
0
20000
40000
60000
Experimental scan number
C
Distributions of residuals from best−fit line
of predicted RT vs Expt. scan number
Line: Z score of novel peptide
Z: 1.73
count
1000
750
500
250
0
−10
−5
0
5
10
Z scores of residuals from best linear fit
